# Recent infection with HCoV-OC43 may be associated with protection against SARS-CoV-2 infection

**DOI:** 10.1016/j.isci.2022.105105

**Published:** 2022-09-09

**Authors:** A.H. Ayesha Lavell, Jonne J. Sikkens, Arthur W.D. Edridge, Karlijn van der Straten, Ferdyansyah Sechan, Melissa Oomen, David T.P. Buis, Michiel Schinkel, Judith A. Burger, Meliawati Poniman, Jacqueline van Rijswijk, Menno D. de Jong, Godelieve J. de Bree, Edgar J.G. Peters, Yvo M. Smulders, Rogier W. Sanders, Marit J. van Gils, Lia van der Hoek, Marije K. Bomers

**Affiliations:** 1 Amsterdam UMC Location Vrije Universiteit Amsterdam, Department of Internal Medicine, De Boelelaan 1117, 1081 HV, Amsterdam, the Netherlands; 2 Amsterdam Institute for Infection and Immunity, Amsterdam, the Netherlands; 3 Amsterdam UMC Location University of Amsterdam, Department of Medical Microbiology and Infection Prevention, Laboratory of Experimental Virology, Meibergdreef 9, 1105 AZ, Amsterdam, the Netherlands; 4 Amsterdam UMC Location University of Amsterdam, Department of Internal Medicine, Meibergdreef 9, 1105 AZ, Amsterdam, the Netherlands; 5 Center for Experimental and Molecular Medicine (CEMM), Amsterdam UMC Location Academic Medical Center, Meibergdreef 9, 1105 AZ, Amsterdam, the Netherlands; 6 Department of Microbiology and Immunology, Weill Medical College of Cornell University, New York, NY 10021, USA

**Keywords:** Molecular physiology, Immunology, Virology

## Abstract

Antibodies against seasonal human coronaviruses (HCoVs) are known to cross-react with SARS-CoV-2, but data on cross-protective effects of prior HCoV infections are conflicting. In a prospective cohort of healthcare workers (HCWs), we studied the association between seasonal HCoV (OC43, HKU1, 229E and NL63) nucleocapsid protein IgG and SARS-CoV-2 infection during the first pandemic wave in the Netherlands (March 2020 – June 2020), by 4-weekly serum sampling. HCW with HCoV-OC43 antibody levels in the highest quartile, were less likely to become SARS-CoV-2 seropositive when compared with those with lower levels (6/32, 18.8%, versus 42/97, 43.3%, respectively: p = 0.019; HR 0.37, 95% CI 0.16–0.88). We found no significant association with HCoV-OC43 spike protein IgG, or with antibodies against other HCoVs. Our results indicate that the high levels of HCoV-OC43-nucleocapsid antibodies, as an indicator of a recent infection, are associated with protection against SARS-CoV-2 infection; this supports and informs efforts to develop pancoronavirus vaccines.

## Highlights

• High OC43 anti-nucleocapsid IgG suggests a recent infection with human coronavirus OC43
• Persons with high OC43 anti-nucleocapsid IgG are less likely to contract SARS-CoV-2

## Graphical abstract

**Figure:**
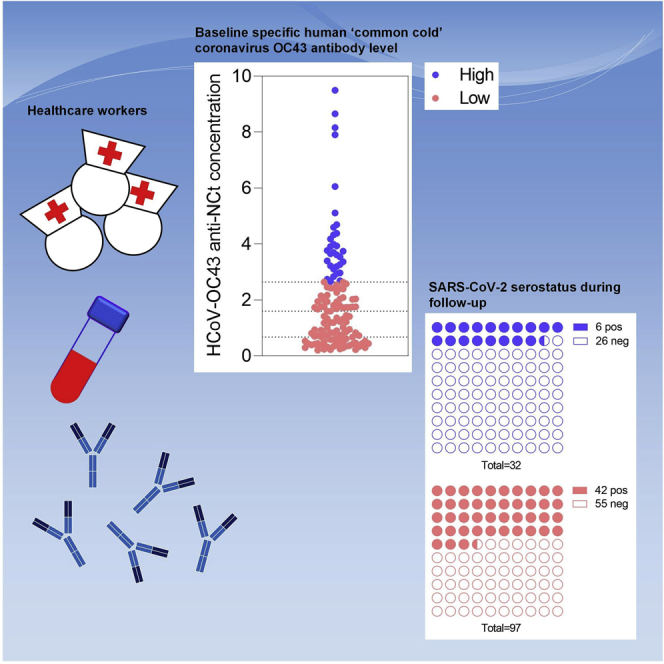

## Introduction

The ongoing SARS-CoV-2 pandemic is characterized by a large individual variability in the risk of contracting infection and subsequent disease severity (Hu et al., 2021; Liu, 2021). Vaccination efforts have been successful in protecting individuals against symptomatic infection and especially severe disease, but sustaining long term protection remains a problem, especially in the light of emerging immune-evasive variants (Hoffmann et al., 2022; Lin et al., 2022b). The potential of cross-protection against SARS-CoV-2 infection elicited by previous infections with seasonal human coronaviruses (HCoVs) is therefore of great interest, but studies have yielded conflicting results (Anderson et al., 2021; Dugas et al., 2021; Ladner et al., 2021; Lin et al., 2022a; Ortega et al., 2021; Sagar et al., 2021; Song et al., 2021).

In this study we prospectively followed a cohort of health care workers (HCW) with different levels of exposure to SARS-CoV-2, and assessed the association between levels of pre-existing HCoV antibodies, incidence of SARS-CoV-2 infection over time, disease severity and SARS-CoV-2 neutralizing immunity in those that became infected. Higher baseline HCoV-OC43 nucleocapsid protein IgG concentrations are associated with markedly lower incidence of SARS-CoV-2 infection. Future interventions against coronaviruses could take advantage of this cross-protective effect, e.g., by incorporating conserved coronavirus antigens to generate pancoronavirus vaccines.

## Results

### High HCoV-OC43 nucleocapsid IgG levels are associated with lower SARS-CoV-2 incidence

**Figure 1:**
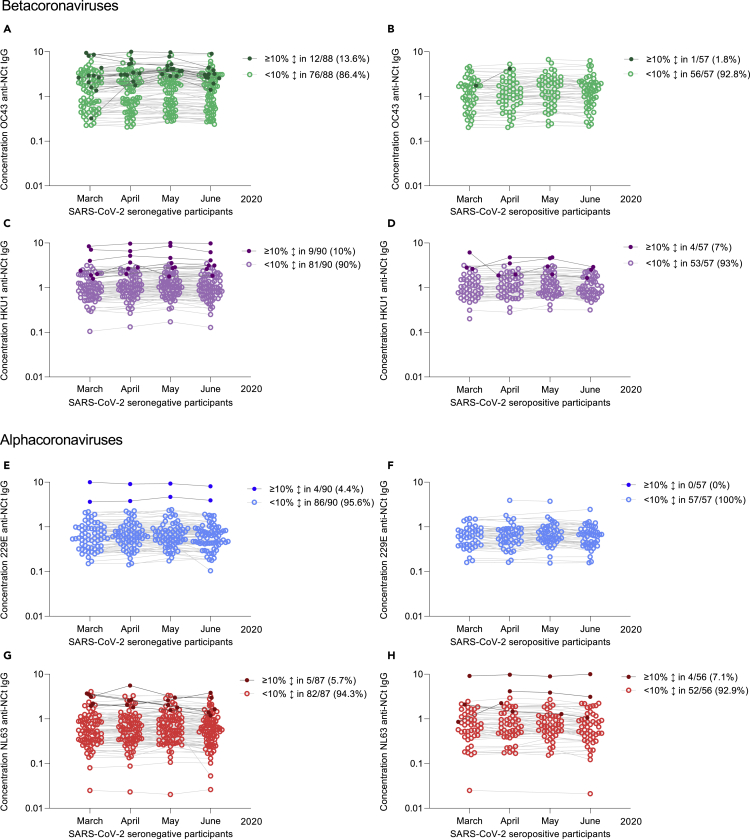
HCoV anti-NCt IgG over time in SARS-CoV-2 seronegative and seropositive participants (A–H) Scatter plots of HCoV IgG against C-terminal nucleocapsid protein (NCt) concentrations over time. To determine fluctuation in antibody concentration, we calculated the difference between the highest and lowest concentration of each participant. A 10% difference in concentration equals 1 unit in standardized antibody concentration.

**Figure 2:**
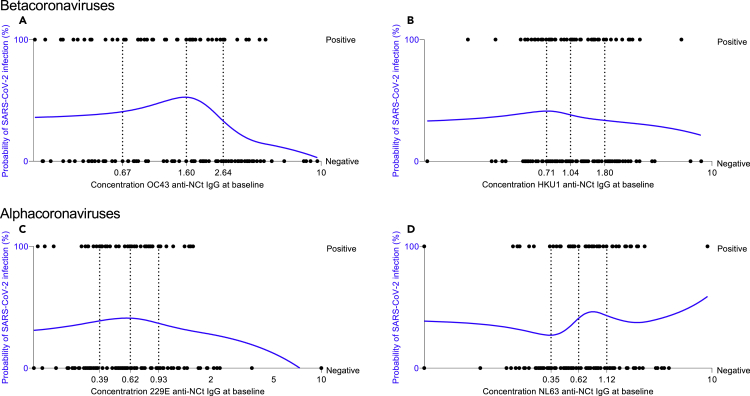
Different levels of HCoV anti-NCt IgG and the probability of SARS-CoV-2 infection (A–D) Plots comparing seasonal HCoV IgG against C-terminal nucleocapsid protein (NCt) concentrations (x-axis) against SARS-CoV-2 status (right y-axis), with fitted binomial spline model with four knots represented by blue line; indicating the probability of seroconversion against SARS-CoV-2 (left y-axis). For further analysis, concentrations of HCoV anti-NCt IgG were divided into quartiles (represented by dotted vertical lines on x-axes).

**Figure 3:**
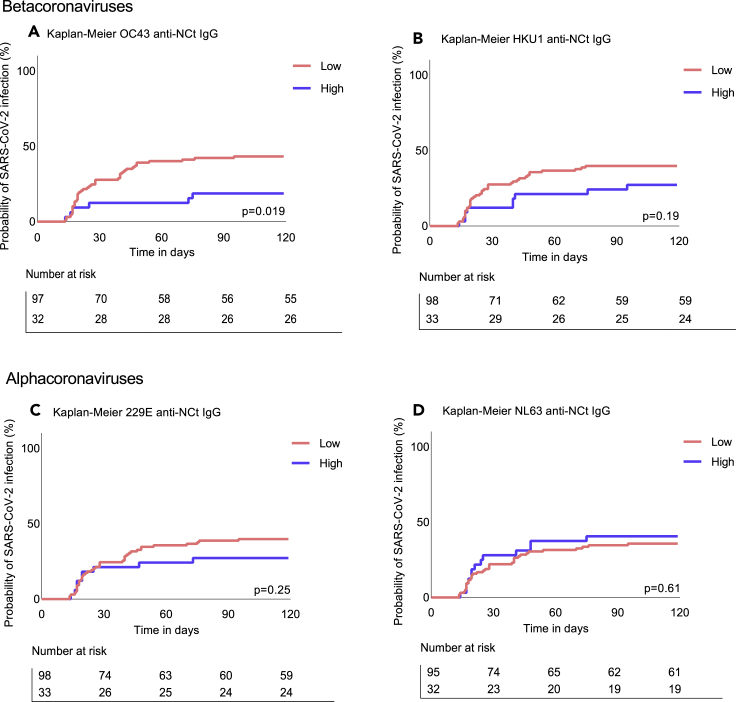
Different levels of baseline HCoV anti-NCt IgG and the incidence of SARS-CoV-2 infection The figure displays Kaplan-Meier survival curves of participants with highest quartile (high) HCoV IgG against C-terminal nucleocapsid protein (NCt) concentrations (red) and those with lower (low) HCoV anti-NCt IgG concentrations (blue), and the incidence of SARS-CoV-2-infection.

**Figure 4:**
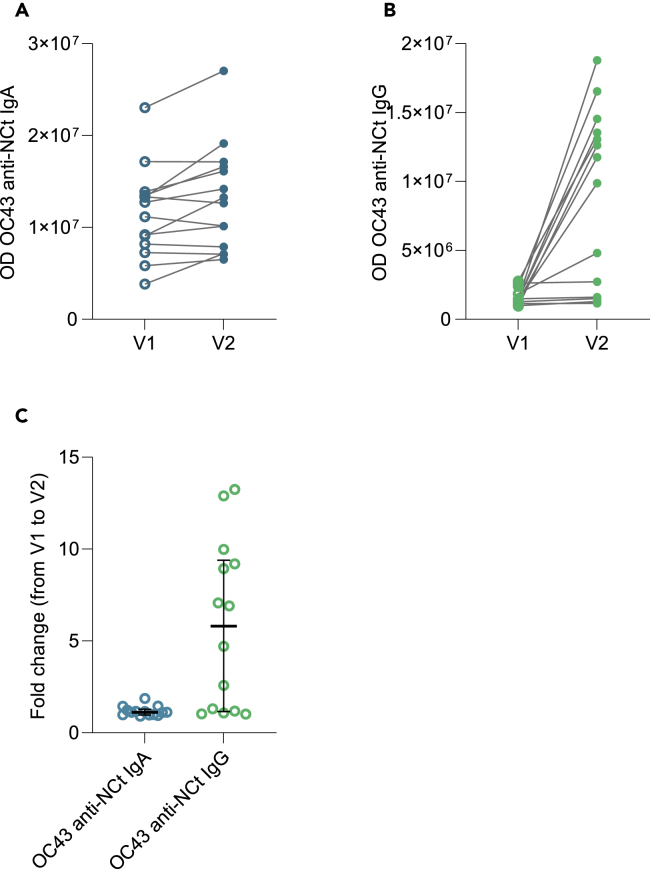
HCoV-OC43 anti-NCt IgA and IgG during and after acute infection HCoV-OC43 anti-NCt IgA (A) and IgG (B) optical density (OD) in ELISA determined in sera of 14 participants from the GRACE observational study (Edridge et al., 2020; Ieven et al., 2018). V1: serum collected during the acute phase of HCoV-OC43-infection, V2: serum collected 28–35 days later. (C) OD fold change from V1 to V2 for IgA and IgG, individual values plotted with median and IQR.

Serum IgG antibodies against the C-terminal domain of nucleocapsid protein (NCt) of seasonal HCoVs OC43, HKU1, 229E, NL63, and total Ig antibodies against S1-RBD of SARS-CoV-2, were measured every 4 weeks during the first COVID-19 wave in the Netherlands (March 2020 - June 2020) in a cohort of 150 HCW (see Table 1 for characteristics). IgG concentrations against all seasonal HCoVs remained relatively stable during the study period (Figures 1A–1H). We hypothesized that if there was any cross-protection by HCoV immunity, this would most likely affect HCW with the most recent seasonal HCoV infection, and therefore those with the highest IgG levels. Plotting the HCoV anti-NCt IgG levels against the probability of contracting a SARS-CoV-2 infection revealed that these potential associations were likely non-linear (Figures 2A–2D). We therefore used baseline seasonal HCoV antibody concentration as a dichotomous determinant throughout the study (highest quartile versus lower concentrations; see Tables S1 and S2). During follow-up, 18.8% (6/32) of participants with anti-NCt IgG concentrations against HCoV-OC43 in the highest quartile at baseline became SARS-CoV-2 seropositive, compared with 43.3% (42/97) of those with lower antibody concentrations (p = 0.019; HR 0.37, 95% CI 0.16–0.88; Figure 3A and Table 2). To correct for possible confounding effects by work-related bedside exposure to COVID-19 patients, we performed a multivariable Cox regression analysis, which showed a consistent result (HR 0.41, 95% CI 0.18–0.97, Table 2). We did not find an association between SARS-CoV-2 infection and anti-NCt IgG levels against HCoV-HKU1, HCoV-229E and HCoV-NL63 (Figures 3B–3D and Table 2). To justify the use of baseline HCoV anti-NCt IgG levels, rather than the antibody levels at each measurement, we performed a sensitivity analysis by using a time-varying determinant in the Cox regression analysis, which results mirrored the earlier found association between HCoV-OC43 IgG concentration and SARS-CoV-2 incidence (HR 0.48, 95% CI 0.23–1.00; Table 2). We did not find an association between SARS-CoV-2 infection and HCoV-OC43 anti-NCt IgA levels (Table 2 and Figures S1A–S1D). Serum-IgA is regarded as one of the earliest markers of infection, yet is only moderately elevated during the first weeks following infection, and therefore a less sensitive marker for recent infection than serum-IgG (Figure 4) (Callow et al., 1990).

**Table 1:** Baseline characteristics

	SARS-CoV-2 seronegative (n = 90)	SARS-CoV-2 seropositive (n = 60)
Median age in years (IQR)	36 (27–50)	32 (27–45)
Sex, women (%)	72 (80.0%)	47 (81.0%)
Work-related exposure		
Bedside COVID-19 patient care	50 (55.6%)	53 (88.3%)
No patient care	40 (44.4%)	7 (11.7%)
Living with children <12 years of age (%)	10 (17.5%)	13 (27.1%)
Disease severity (%)		
No symptoms	–	23 (38.3%)
Any symptoms^a^	–	36 (60.0%)
Minimal		20 (33.3%)
Mild		8 (13.3%)
Moderate		8 (13.3%)
Severe		0
Unknown		1 (1.7%)
First positive (%)		
March 2020	–	31 (51.7%)
April 2020	–	20 (33.3%)
May 2020	–	5 (8.3%)
June 2020	–	4 (6.7%)
SARS-CoV-2 PCR positive (%)		26 (43.3%)

Table showing the baseline characteristics of participants, becoming seropositive and remaining seronegative for SARS-CoV-2 during follow-up.

a Any symptoms are divided into minimal (i.e., without limitations in daily functioning), mild (i.e., some limitations in daily functioning), moderate (i.e., most of the day supine) and severe (i.e., requiring hospital admission).

**Table 2:** Association between baseline HCoV anti-NCt and the incidence of SARS-CoV-2

	Low	High	Log rank p value	Univariable HR (95% CI)	Multivariable HR (95% CI)	Time-varying HR (95% CI)
HCoV anti-NCt IgG						
OC43	42/97 (43.3%)	6/32 (18.8%)	0.019	0.37 (0.16–0.88)	0.41 (0.18–0.97)	0.48 (0.23–1.00)
HKU1	39/98 (39.8%)	9/33 (27.3%)	0.19	0.62 (0.30–1.28)	0.64 (0.31–1.33)	0.65 (0.33–1.30)
229E	39/97 (40.2%)	9/33 (27.3%)	0.25	0.66 (0.32–1.36)	0.67 (0.32–1.38)	0.85 (0.44–1.65)
NL63	34/95 (35.8%)	13/32 (40.6%)	0.61	1.18 (0.62–2.24)	1.12 (0.59–2.12)	1.33 (0.74–2.37)
HCoV anti-NCt IgA						
OC43	33/96 (34.4%)	14/32 (43.8%)	0.40	1.31 (0.70–2.44)	1.15 (0.61–2.16)	1.21 (0.67–2.19)

Univariable survival and Cox regression analysis showing the association between the highest quartile (high) versus lower quartiles (low) of IgG concentrations against C-terminal nucleocapsid protein (NCt) of HCoVs at baseline (as a dichotomous determinant) and IgA against NCt of HCoV-OC43, and incidence of SARS-CoV-2 infection. Multivariable analysis is corrected for work-related exposure to COVID-19 patients. Time-varying Cox regression analysis shows the same association between HCoV anti-NCt concentration and SARS-CoV-2 incidence, but using the HCoV concentration at each measurement (rather than only at baseline) as determinant for the preceding time-interval.

### Influenza- and RSV- antibodies are not associated with SARS-CoV-2 incidence

To support the conclusion of a HCoV-OC43- or betacoronavirus-specific protective effect we examined the presence of cross-immunity induced by non-coronavirus respiratory viruses; seasonal influenza virus and respiratory syncytial virus (RSV). The results did not indicate a significant difference in SARS-CoV-2 incidence for those with high seasonal influenza virus hemagglutinin (HA)- or RSV fusion protein (RSV-F)-antibody concentrations compared with those with low antibody concentrations (14/33, 42.4%, versus 34/97, 35.1%, p = 0.42; HR 1.29, 95% CI 0.69–2.40, and 13/33, 39.4%, versus 35/97, 36.1%, p = 0.68; HR 1.44, 95% CI 0.61–2.16, respectively; Table S3).

### HCoV nucleocapsid IgG did not affect severity of SARS-CoV-2 infection or neutralizing capacity

None of the participants in our cohort reported severe COVID-19 disease requiring hospital admission. Asymptomatic SARS-CoV-2-infection was reported by 18 (37.5%) of the 48 seropositive participants, and 29 (60.4%) reported symptoms varying from minimal to moderate. For one participant the severity of symptoms was not reported. We found no clear association between baseline high or low HCoV-OC43 anti-NCt IgG concentrations and presence of any symptoms during SARS-CoV-2 infection (OR 0.68, 95% CI 0.17–2.76; Table 3), even after adjustment for possible confounding by age and sex (OR 0.28, 95% CI 0.05–1.38; Table 3). We also found no association between HCoV-HKU1, HCoV-229E, and HCoV-NL63 anti-NCt IgG concentrations, and COVID-19 disease severity (Table 3).

**Table 3:** Association between baseline HCoV anti-NCt IgG and severity of SARS-CoV-2

HCoV anti-NCt IgG	Univariable OR (95% CI)	Multivariable OR (95% CI)
OC43	0.68 (0.17–2.76)	0.28 (0.05–1.38)
HKU1	0.52 (0.13–2.00)	0.71 (0.16–3.33)
229E	1.33 (0.35–5.78)	0.68 (0.14–3.33)
NL63	1.40 (0.36–6.09)	2.31 (0.51–13.18)

Table showing the results of logistic regression to test for the association between the highest quartile versus lower quartiles of anti-NCt IgG levels against seasonal HCoVs at baseline (as a dichotomous determinant) and severity of SARS-CoV-2 infection defined as asymptomatic or symptomatic. Multivariable analysis is corrected for sex and age.

To examine the association between baseline seasonal HCoV anti-NCt IgG concentration and neutralizing capacity against SARS-CoV-2 in infected individuals, we measured SARS-CoV-2 neutralizing capacity of sera collected on the fourth and last measurement in SARS-CoV-2 seroconverted individuals using a pseudovirus based neutralization assay. Neutralizing capacity was not associated with high or low baseline HCoV-OC43 anti-NCt IgG concentrations (mean log ID50 5.64 versus 5.27, difference −0.37 log ID50, 95% CI -1.23–0.49; Table 4 and Figure S2A). Results were similar for HCoV-HKU1, HCoV-229E and HCoV-NL63 (Table 4 and Figures S2B–S2D).

**Table 4:** Association between baseline HCoV anti-NCt and neutralizing capacity (log ID_50_) against SARS-CoV-2

HCoV anti-NCt IgG	Univariable			Multivariable
	Mean log ID50 (lowest quartiles	Mean log ID50 (highest quartile	Difference high versus low (95% CI)	Difference high versus low (95% CI)
OC43	5.64	5.27	−0.37 (−1.23–0.49)	−0.41 (−1.28–0.47)
HKU1	5.49	5.73	0.25 (−0.59–1.09)	0.23 (−0.63–1.08)
229E	5.75	4.99	−0.76 (−1.58–0.05)	−0.74 (−1.57–0.10)
NL63	5.50	5.78	0.28 (−0.57–1.12)	0.29 (−0.57–1.14)

Results of linear regression analysis for the association between highest quartile versus lower quartiles of antibody levels against HCoV C-terminal of nucleocapsid protein (NCt) and SARS-CoV-2 neutralizing capacity (measured in June 2020) in log ID_50_ in SARS-CoV-2 seropositive participants. Multivariable analysis is corrected for time to infection with SARS-CoV-2.

### Seasonal HCoV IgG levels against spike protein are not associated with SARS-CoV-2 incidence

To explore why previous studies using seasonal HCoV spike (S) IgG found no evidence for cross-immunity, we replicated our analysis with anti-S (rather than anti-NCt) IgG. There was substantial variation in HCoV anti-S IgG compared with HCoV anti-NCt IgG over time. This was comparable for those with and without SARS-CoV-2 infection during the study period (Figures S3A–S3H). We found no significant difference in the incidence of SARS-CoV-2 infection between individuals in the highest quartile of baseline HCoV-OC43 S-antibody concentrations and those with lower concentrations (10/33, 30.3%, versus 38/98, 38.8%, p = 0.33; HR 0.71, 95% 0.35–1.42; see Table S4). We considered whether cross-reactivity of HCoV anti-S IgG boosted by SARS-CoV-2 infection could mask a potential relation between HCoV anti-S IgG and SARS-CoV-2 infection. Therefore, we repeated the analysis including only the samples in which HCoV anti-S IgG were measured 4 weeks before SARS-CoV-2 seropositivity, which yielded comparable results (4/27, 14.8%, versus 13/73, 17.8%, p = 0.68 for participants with highest quartile of baseline HCoV-OC43 anti-S IgG concentrations versus lower baseline HCoV-OC43 anti-S IgG concentrations; HR 0.79, 95% CI 0.26–2.41, Table S4). Similarly, no significant difference in the incidence of SARS-CoV-2 infection was detected in individuals with highest quartile versus lower anti-S IgG concentrations of HCoV-HKU1, HCoV-229E and HCoV-NL63 with either analysis method (see Table S4).

### Seasonal HCoV nucleocapsid IgG levels are not associated with SARS-CoV-2 spike or RBD IgG concentration in COVID naive individuals

**Figure 5:**
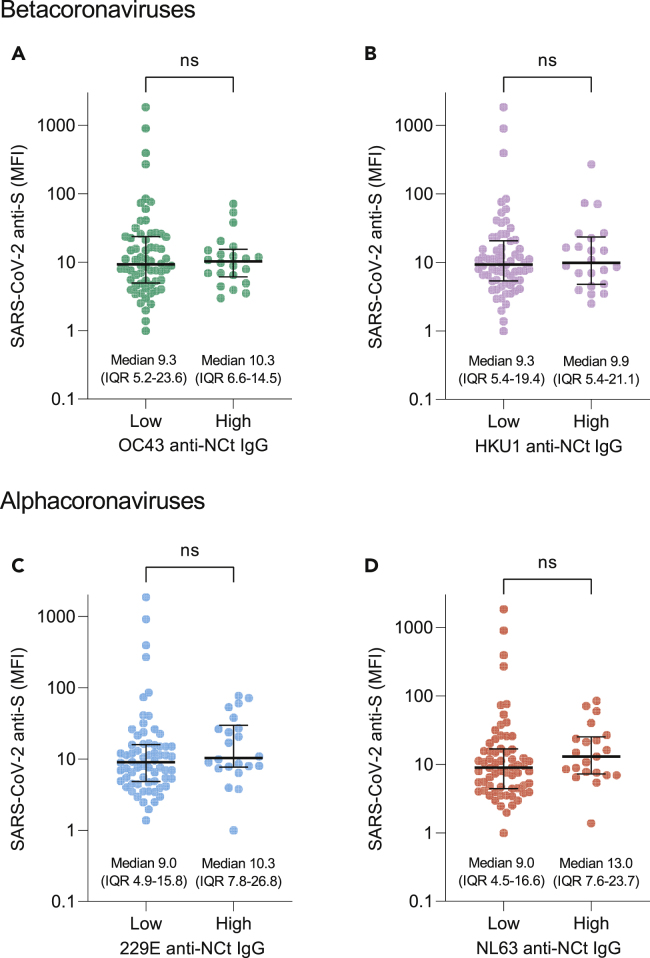
SARS-CoV-2 anti-S IgG in COVID naive individuals (A-D) Comparison of IgG against SARS-CoV-2 spike (S) in MFI between participants with highest quartile (high) seasonal HCoV anti-C-terminal nucleocapsid protein (NCt) IgG levels against lower (low) three quartiles at baseline. Participants were SARS-CoV-2 negative at baseline and the second measurement. Data are represented as individual values, median and interquartile range (IQR). ns: not significant, assessed by Mann–Whitney U test.

**Figure 6:**
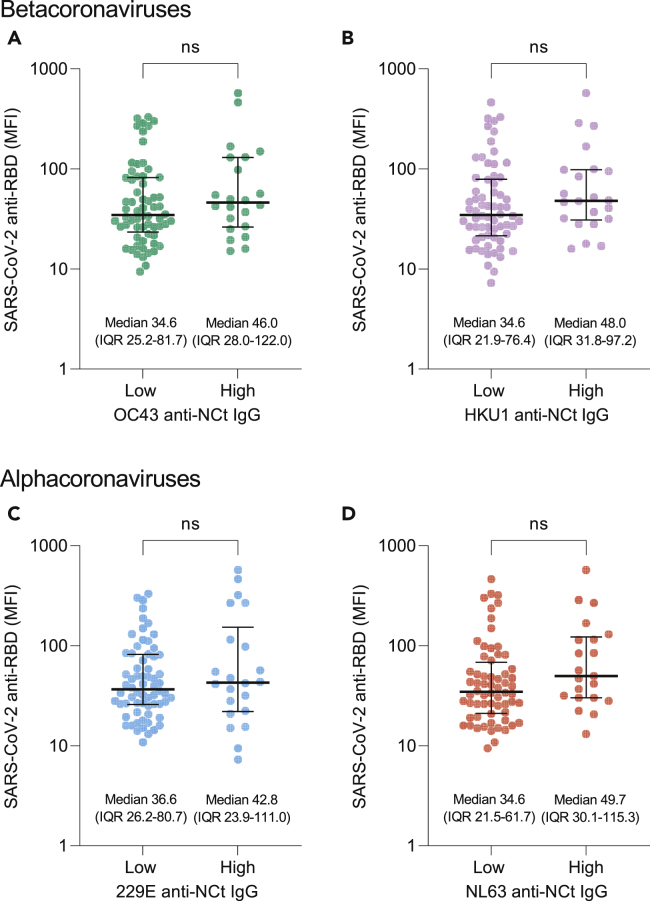
SARS-CoV-2 anti-RBD IgG in COVID naive individuals (A-D) Comparison of antibodies against SARS-CoV-2 receptor-binding domain (RBD) in MFI between participants with highest quartile (high) seasonal HCoV anti-C-terminal domain of nucleocapsid protein (NCt) IgG levels against lower (low) three quartiles at baseline. Participants were SARS-CoV-2 negative at baseline and the second measurement. Data are represented as individual values, median and interquartile range (IQR). ns: not significant, assessed by Mann–Whitney U test.

We hypothesized that if HCoV-OC43 cross-immunity against SARS-CoV-2 infection is mediated via SARS-CoV-2 neutralization, one would expect higher concentrations of SARS-CoV-2 spike (S) or receptor-binding domain (RBD) IgG in individuals with high HCoV-OC43 anti-NCt IgG in SARS-CoV-2 naive individuals. However, there was no difference between participants with high versus low levels of HCoV-OC43 anti-NCt IgG and SARS-CoV-2 anti-S IgG levels (median 10.3 MFI, IQR 6.6–14.5, versus median MFI 9.3, IQR 5.2–23.6, p = 0.91, respectively; Figure 5) or SARS-CoV-2 anti-RBD IgG levels (median 46.0 MFI, IQR 28.0–122.0, versus median 34.6 MFI, IQR 25.2–81.7, p = 0.24, respectively; Figure 6).

## Discussion

In a prospective cohort of HCWs followed during the first wave of the SARS-CoV-2 pandemic, we found that for individuals with high baseline HCoV-OC43 NCt-IgG levels, the probability of SARS-CoV-2 infection was substantially reduced. IgG antibody concentrations steadily decline over time, therefore high antibody levels suggest a recent HCoV infection (Callow et al., 1990; Edridge et al., 2020). The effect was robust for adjustment for COVID-19 exposure; this association was not found for HCoV-OC43 anti-NCt IgA and anti-S IgG, nor for antibodies against other HCoVs, influenza virus and RSV.

Our findings complement the growing body of evidence that pre-existing immunity to seasonal HCoV can protect against SARS-CoV-2 (Braun et al., 2020; Kundu et al., 2022; Ladner et al., 2021; Mateus et al., 2020; Ortega et al., 2021; Sagar et al., 2021; Schulien et al., 2021; Song et al., 2021), although results have not been consistent throughout previous studies. Higher levels of nucleocapsid-antibodies against HCoV-OC43 and HCoV-HKU1 were associated with a less severe course of COVID-19, and lower levels with a higher rate of intensive care admissions (Dugas et al., 2021). In line with our findings, a previous study observed a trend towards higher HCoV N-antibody levels at baseline in HCW who subsequently did not become infected with SARS-CoV-2 (Ortega et al., 2021). On the contrary, two previous studies examining the correlation between baseline HCoV antibodies and protection against SARS-CoV-2 infection in longitudinally sampled populations, did not find the protective effect we describe (Anderson et al., 2021; Lin et al., 2022a). Authors concluded pre-existing betacoronavirus antibodies may actually negatively impact protection, because higher magnitudes correlate with more SARS-CoV-2 antibodies following infection, as a proxy for greater disease severity (Lin et al., 2022a). The use of different antibody targets (i.e. spike or nucleocapsid protein) may explain these discordant results. The C-terminal domain of nucleocapsid protein we used in our ELISA, is carefully chosen because this part of the viral protein is well preserved within, but less conserved between HCoV species (Dijkman et al., 2008; Edridge et al., 2020; Leach et al., 2021). The specificity of this test is 100% and sensitivity 97%, therefore, the detected antibodies against NCt are unlikely to be cross-reactive (Edridge et al., 2020). In contrast, when one uses the full-length version of the S protein in a serological test the specificity is likely reduced, because antibodies against epitopes located in the S2-subunit are known to be more reactive across species (Grobben et al., 2021; Ladner et al., 2021). The SARS-CoV-2 infection-induced antibodies that cross-react with the S for seasonal coronaviruses, may mask the fact that those not infected by SARS-CoV-2 had higher antibodies recognizing HCoV-OC43 S to start with. Furthermore, HCoV immunity is not long-lasting (Edridge et al., 2020); differences in interval between baseline HCoV antibody sampling and SARS-CoV-2 exposure observed between previous studies and this one, may further contribute to different outcomes.

Sera of SARS-CoV-2 uninfected individuals that contained cross-reacting antibodies were found to have neutralizing potential which could contribute to protection against SARS-CoV-2 (Galipeau et al., 2021; Ng et al., 2020; Song et al., 2021), although several others did not detect cross-neutralizing capacity (Anderson et al., 2021; Poston et al., 2021; Selva et al., 2021). N-antibodies lack neutralizing potential, but may intrinsically contribute to immune response e.g. by interfering with complement activation (Galipeau et al., 2021; Kang et al., 2021). Boosting of IgA in the upper airway mucosa is described as another pathway by which humoral immunity acquired by recent heterologous HCoV infection may add to cross-protection (Callow, 1985; Cervia et al., 2021; Russell et al., 2020).

We did not demonstrate an association between baseline HCoV-OC43 anti-NCt IgG and decreased SARS-CoV-2 severity, nor did we detect signs of antibody dependent enhancement of disease, as suggested by others (Arvin et al., 2020; Huang et al., 2020; Lin et al., 2022a). In addition, we did not find an association between baseline seasonal HCoV anti-NCt IgG and neutralization capacity after SARS-CoV-2 infection or SARS-CoV-2 S- or RBD-antibody concentration (as a surrogate for potential neutralization capacity) in SARS-CoV-2 uninfected individuals. We also did not find a protective association for anti-NCt IgG concentrations of betacoronavirus HCoV-HKU1 and alphacoronaviruses HCoV-NL63 and HCoV-229E. The latter might be explained by the decreased homology between alpha- and betacoronaviruses including SARS-CoV-2 (Huang et al., 2020; Liu, 2021; Song et al., 2021). The less robust antibody response following HCoV-HKU1 infection, with difficulty to recognize recent infection by this virus, may explain the lack of detection of a protective association with HCoV-HKU1 anti-NCt IgG (Edridge et al., 2020; Sechan et al., 2022).

As described above, humoral cross-immunity may mediate protection in itself. Alternatively, protection against SARS-CoV-2 could be explained by cross-reactive cellular immunity (Braun et al., 2020; Grifoni et al., 2020; Le Bert et al., 2020; Lineburg et al., 2021; Mateus et al., 2020; Schulien et al., 2021), with HCoV antibody levels being merely a marker for recent infection; and both explanations are not mutually exclusive. Kundu et al. recently reported on household contacts recruited shortly after exposure to COVID-19 patients (Kundu et al., 2022). Baseline N-targeting antibodies against seasonal HCoVs were associated with both higher frequencies of cross-reactive T cells, and not contracting SARS-CoV-2 infection during follow-up. Contacts who remained SARS-CoV-2 negative during follow-up showed significantly higher frequencies of specific IL-2 secreting memory T cells that cross-react with HCoV, compared to contacts who became SARS-CoV-2 positive. Cross-reactive T cells were depleted from the bloodstream within days to weeks after exposure, suggesting migration from the circulation to the affected respiratory mucosa (Kundu et al., 2022). Similarly, another study demonstrated that closely (NAAT and serologically) monitored HCW who did not contract SARS-CoV-2 infection despite exposure, had stronger, more multispecific memory T cells, compared with an unexposed pre-pandemic cohort, with expansion of T cells able to cross recognize shared HCoV epitopes (Swadling et al., 2021).

An important strength of this study is the prospectively collected, longitudinal data on seasonal HCoV immunity with detailed surveillance of SARS-CoV-2 incidence. The study period comprises the very first pandemic months of the Netherlands, with fresh immunologic memory of endemic HCoV infections, which is not yet hampered by SARS-CoV-2 preventive measures. The 4-week interval of serum sampling allows us to interpret dynamics of HCoV antibody levels, rather than solely depend on cross-sectional antibody concentrations. Our study also benefits from the use of a highly specific HCoV anti-NCt assay.

In conclusion, this study found that HCW with high IgG antibodies against HCoV-OC43 NCt were less frequently infected with SARS-CoV-2. We corroborate that immunity induced by one HCoV infection can confer short-lived protection against another HCoV. Downscaling strict SARS-CoV-2 preventive measures will likely be accompanied by recurrence of endemic HCoV infections. Cross-protection derived from HCoV might, at least partially, contribute in controlling the SARS-CoV-2 pandemic, and vaccine development may benefit from incorporating antigens which are conserved between coronavirus species to attempt to generate pancoronavirus immunity.

### Limitations of the study

The study is limited by the relatively small sample size. The width of several 95% confidence intervals, e.g. for the association between HCoV-OC43 anti-NCt IgG concentration and SARS-CoV-2 severity (OR 0.68, 95% CI 0.17–2.76, or 0.28, 95% CI 0.05–1.38 after correction for sex and age), suggests the study may have been underpowered to detect clinically relevant associations. Also, none of the relatively young and healthy participants suffered from severe COVID-19. The generalizability of our findings to the current era of emerging variants of concern is uncertain.

## STAR★Methods

### Key resources table

**Table:** 

REAGENT or RESOURCE	SOURCE	IDENTIFIER
**Biological Samples**		

Human sera from HCW	Amsterdam UMC	N/A
Human sera from patients (GRACE)	Ieven et al. (2018)	N/A

**Chemicals, Peprtides and Recombinant/Viral Proteins**		

HCoV-OC43 C-terminal domain nucleocapsid	Edridge et al. (2020)	N/A
HCoV-HKU1 C-terminal domain nucleocapsid	Edridge et al. (2020)	N/A
HCoV-OC43 C-terminal domain nucleocapsid	Edridge et al. (2020)	N/A
HCoV-NL63 C-terminal domain nucleocapsid	Edridge et al. (2020)	N/A
HCoV-OC43 spike	GenBank	AAT84362.1
HCoV-HKU1 spike	GenBank	Q0ZME7
HCoV-229E spike	GenBank	NP_073551.1
HCoV-NL63 spike	GenBank	AKT07952.1
Respiratory syncytial virus fusion glycoprotein	McLellan et al. (2013)	N/A
Influenza A/H1N1pdm09 virus HA protein	Aartse et al. (2021)	N/A

**Critical Commercial Assays**		

Wantai SARS-CoV-2 Ab ELISA	Beijing Wantai Biological Pharmacy Enterprise Co.	WS-1096
HCoV ELISA	Edridge et al. (2020)	N/A
MAGPIX	Luminex	MAGPIX-XPON4.1-RUO
Luminex Magplex beads	Luminex	MC10043-01
Nano-Glo Luciferase Assay System	Promega	Cat# N1130
GloMax	Turner BioSystems	Cat# 9101-002

**Experimental Models:Cell Lines**		

HEK293T/ACE2 cells	Schmidt et al. (2020)	RRID: CVCL_A7UK
FreeStyle HEK293F cells	Thermo Fisher	RRID: CVCL_D603
HEK293T cells	ATCC	Cat# CRL-11268

**Antibodies**		

Alkaline phosphatase-conjugated AffiniPure Goat Anti-Human IgG, Fc Fragment Specific	Jackson ImmunoResearch	CAT# 109–055–170; RRID: AB_2810893
Peroxidase AffiniPure F(ab')₂ Fragment Goat Anti-Human Serum IgA, α chain specific	Jackson ImmunoResearch	CAT# 109-036-011; RRID: AB_2337592
Goat-anti-human IgG-PE (goat polyclonal)	Southern Biotech	RRID: AB_2795648

**Other**		

Carbonate-Bicarbonate Buffer Capsule	Sigma-Aldrich	C3041-50CAP
PBS tablets	Gibco	18912014
Tween-20	Sigma-Aldrich	9005-64-5
Nonfat dried milk powder,1 kg	AppliChem	APA0830.1000
Lumi-Phos Plus	Lumigen	P-7000
Lumi-Phos HRP	Lumigen	PSA-1000
Water, HPLC, J.T.Baker™	Fisher Scientific	14-650-357
1-Ethyl-3-(3-dimethylaminopropyl) carbodiimide	Thermo Fisher Scientific	Cat#:A35391
Sulfo-N-hydroxysulfosuccinimide	Thermo Fisher Scientific	Cat#:A39269
Luminex Magplex beads	Luminex	MC10043-01

**Software and Algorithms**		

R version 4.0.3	R	N/A
Graphpad Prism version 9	Graphpad	N/A
GloMax Navigator	Promega	N/A

### Resource availability

#### Lead contact

Further information and requests for resources should be directed to the lead contact, Marije Bomers (m.bomers@amsterdamumc.nl).

#### Materials availability

This study did not generate new unique reagents.

### Experimental model and subject details

#### Study design and participants

In March 2020, the first month of the SARS-CoV-2 pandemic in the Netherlands, we started a prospective serologic surveillance cohort study among hospital HCWs in the Amsterdam University Medical Center (UMC), consisting of two tertiary care hospitals (S3 study) (Sikkens et al., 2021). Participants underwent phlebotomies combined with surveys regarding exposure to COVID-19 patients, presence of COVID-19 related symptoms, and results of nucleic acid amplification testing (NAAT). All participants were assumed to be seronegative on the day the first SARS-CoV-2 infection was established in the Netherlands (February 27, 2020). Follow-up visits were scheduled every 4 weeks over 18 weeks during the first wave (started March 23, 2020 and finished on June 25, 2020). The first patient with confirmed COVID-19 was admitted to the Amsterdam UMC on March 9, 2020.

Within this cohort we compared the 60 HCW that contracted SARS-CoV-2 infection during follow-up to a group of 90 seronegative HCW. The latter were selected on work-related COVID-19 exposure and highest attendance to follow-up visits. Work-related exposure was defined as working in direct patient care with COVID-19 patients (e.g., intensive care unit, emergency department or a dedicated COVID-19 ward) versus not working in patient care.

The study was approved by the medical ethics review committee of both hospitals, and written informed consent was obtained from each participant. More comprehensive details about the original S3 cohort have been published previously (Sikkens et al., 2021).

### Method details

#### Serological tests

SARS-CoV-2 specific antibodies were determined by measuring total-Ig against S1-RBD using the commercially available Wantai enzyme-linked immunosorbent assay (ELISA) (Zhao et al., 2020). IgG against the C-terminal domain of the nucleocapsid protein (NCt) of the seasonal HCoVs were measured using a previously described ELISA (Dijkman et al., 2008; Edridge et al., 2020). This NCt antibody test detects antibodies only recognizing linear epitopes with a specificity of 100% and sensitivity of 97% (Edridge et al., 2020). IgA against NCt of HCoV-OC43 were measured by ELISA as previously described (Edridge et al., 2020) with three modifications: the sera of participants were diluted 1:50 in PBS and 0.1% Tween-20 (PBST) with 1% nonfat milk (AppliChem); the secondary antibody (Peroxidase AffiniPure F(ab')₂ Fragment Goat Anti-Human Serum IgA, α chain specific, Jackson ImmunoResearch) were diluted 1:5000 in PBST with 1% nonfat milk, and the ELISA signal was developed using Lumi-Phos HRP (Lumigen), diluted 1:10 in HPLC-grade water (J.T.Baker). Optical density signals were calibrated using an 8-step serial dilution of a reference sample per HCoV antigen on each ELISA plate (IgG and IgA test) and converted into arbitrary units. The arbitrary units were standardized by dividing by the highest measured concentration of each seasonal HCoV and multiplying by ten, i.e., each unit difference represents a difference of 10% in concentration.

As a comparison, IgA and IgG against NCt of HCoV-OC43 were determined in sera from 14 participants of the GRACE observational study, collected during the acute phase of a HCoV-OC43-infection (V1) and subsequently 28–35 days after (V2) (Edridge et al., 2020; Ieven et al., 2018).

IgG antibodies against spike proteins of the seasonal HCoVs and against SARS-CoV-2 were determined using the custom Luminex assay. The Luminex assay and the protein design are described previously (Grobben et al., 2021). In short, prefusion stabilized trimeric spike protein ectodomains contained both the S1 and S2-subdomain. More information about the exact cleavage site can be found in (Grobben et al., 2021) for SARS-CoV-2 S and in (Brouwer et al., 2020) for HCoV-OC43, -HKU1, -229E and-NL63. All proteins were covalently coupled to Luminex MagPlex beads with a ratio of 75 mg protein to 12.5 million beads. The seasonal influenza virus hemagglutinin (HA) and the fusion peptide of respiratory syncytial virus (RSV-F) were coupled equimolar to the coronavirus spike proteins. Optimization studies showed an optimal dilution of sera of 1:10,000 for measuring the infection response. After an overnight incubation, plates were washed with TBS containing 0.05% Tween-20 (TBS-Tween) and resuspended in 50 mL of Goat Anti-Human IgG-PE (RRID AB_2795648, validated by SouthernBiotech). Read-out was performed on a Magpix (Luminex). Resulting median fluorescent intensity (MFI) values are the median of approximately 50 beads per well and were background corrected by subtraction of MFI values from buffer and beads-only wells. The S-antibody Luminex-test specificity and sensitivity has not been evaluated but it is expected to be less specific as the protein in this test also contains the more-conserved-S2 region.

#### Pseudovirus neutralization assay

Sera of SARS-CoV-2 infected participants obtained two weeks to four months after infection were analyzed in a pseudovirus neutralization assay, as previously described (Brouwer et al., 2021). Heat-inactivated sera were serially diluted and 1:1 mixed with SARS-CoV-2 pseudovirus. After one hour incubation at 37°C, the mixture was added to HEK293T cells expressing angiotensin converting enzyme 2 (ACE2) receptor and incubated for 48 h at 37°C. Subsequently cells were lysed and luciferase activity was measured using a Nano-Glo Luciferase Assay System (Promega). Relative luminescence units were normalized to the units from cells infected with pseudovirus in absence of serum. Neutralization levels were based on the serum dilution at which infectivity was inhibited 50% (ID_50_). ID_50_ values < 20 were considered as absence of neutralization.

#### Outcomes

SARS-CoV-2 infection was defined as either a positive NAAT result and/or presence of specific SARS-CoV-2 antibodies as detected by the aforementioned Wantai ELISA. The date of SARS-CoV-2 infection was defined as the sampling date of a first positive NAAT result or, in its absence, the midpoint between the last seronegative and the first seropositive sample. All participants that tested positive for SARS-CoV-2 by NAAT during follow-up also developed SARS-CoV-2 specific antibodies in at least one serum sample. The severity of COVID-19 was defined as asymptomatic, minimal (i.e., without limitations in daily functioning) to mild (i.e., with some limitations in daily functioning) or moderate symptoms (i.e., being supine most of the day) and severe disease requiring hospital admission.

### Quantification and statistical analysis

We used Kaplan-Meier estimates and Cox regression analysis to assess time to event outcomes. We report log-rank p-values and hazard ratios with 95% confidence intervals as primary results. When the number of events per subgroup was 5 or less, we only report the p-value of the log-rank test as the primary result. Time to SARS-CoV-2 infection was defined as the elapsed time between the date of the first confirmed SARS-CoV-2 infection in the Netherlands (February 27, 2020) and the date of confirmed SARS-CoV-2 infection. We used logistic regression to compare binary outcomes. The results of Cox regression and logistic regression were considered statistically significant when the 95% confidence interval (CI) did not encompass 1. Continuous outcomes were compared using Mann–Whitney U test or linear regression. Statistical tests were performed in R version 4.0.3. The spline models and corresponding figures were made in GraphPad Prism version 9.

### Additional resources

This study is registered in the Netherlands Trial Register (NL8645). URL: https://trialregister.nl/trial/8645.

## Data Availability

• The data reported in this study cannot be deposited in a public repository because privacy restrictions may apply. To request access to data, contact Marije Bomers (m.bomers@amsterdamumc.nl). In addition, summary statistics describing these data have been deposited in tables and figures of this manuscript and the supplemental material and are publicly available as of the date of publication. • This article does not report original code. • Any additional information required to reanalyze the data reported in this article is available from the lead contact upon request.

## References

[bib1] Aartse A., Eggink D., Claireaux M., van Leeuwen S., Mooij P., Bogers W.M., Sanders R.W., Koopman G., van Gils M.J. (2021). Influenza a virus hemagglutinin trimer, head and stem proteins identify and quantify different hemagglutinin-specific b cell subsets in humans. Vaccines.

[bib2] Anderson E.M., Goodwin E.C., Verma A., Arevalo C.P., Bolton M.J., Weirick M.E., Gouma S., McAllister C.M., Christensen S.R., Weaver J. (2021). Seasonal human coronavirus antibodies are boosted upon SARS-CoV-2 infection but not associated with protection. Cell.

[bib3] Arvin A.M., Fink K., Schmid M.A., Cathcart A., Spreafico R., Havenar-Daughton C., Lanzavecchia A., Corti D., Virgin H.W. (2020). A perspective on potential antibody-dependent enhancement of SARS-CoV-2. Nature.

[bib4] Braun J., Loyal L., Frentsch M., Wendisch D., Georg P., Kurth F., Hippenstiel S., Dingeldey M., Kruse B., Fauchere F. (2020). SARS-CoV-2-reactive T cells in healthy donors and patients with COVID-19. Nature.

[bib5] Brouwer P.J.M., Brinkkemper M., Maisonnasse P., Dereuddre-Bosquet N., Grobben M., Claireaux M., de Gast M., Marlin R., Chesnais V., Diry S. (2021). Two-component spike nanoparticle vaccine protects macaques from SARS-CoV-2 infection. Cell.

[bib6] Brouwer P.J.M., Caniels T.G., van der Straten K., Snitselaar J.L., Aldon Y., Bangaru S., Torres J.L., Okba N.M.A., Claireaux M., Kerster G. (2020). Potent neutralizing antibodies from COVID-19 patients define multiple targets of vulnerability. Science.

[bib7] Callow K.A. (1985). Effect of specific humoral immunity and some non-specific factors on resistance of volunteers to respiratory coronavirus infection. J. Hyg..

[bib8] Callow K.A., Parry H.F., Sergeant M., Tyrrell D.A.J. (1990). The time course of the immune response to experimental coronavirus infection of man. Epidemiol. Infect..

[bib9] Cervia C., Nilsson J., Zurbuchen Y., Valaperti A., Schreiner J., Wolfensberger A., Raeber M.E., Adamo S., Weigang S., Emmenegger M. (2021). Systemic and mucosal antibody responses specific to SARS-CoV-2 during mild versus severe COVID-19. J. Allergy Clin. Immunol..

[bib10] Dijkman R., Jebbink M.F., El Idrissi N.B., Pyrc K., Müller M.A., Kuijpers T.W., Zaaijer H.L., Van Der Hoek L. (2008). Human coronavirus NL63 and 229E seroconversion in children. J. Clin. Microbiol..

[bib11] Dugas M., Grote-Westrick T., Merle U., Fontenay M., Kremer A.E., Hanses F., Vollenberg R., Lorentzen E., Tiwari-Heckler S., Duchemin J. (2021). Lack of antibodies against seasonal coronavirus OC43 nucleocapsid protein identifies patients at risk of critical COVID-19. J. Clin. Virol..

[bib12] Edridge A.W.D., Kaczorowska J., Hoste A.C.R., Bakker M., Klein M., Loens K., Jebbink M.F., Matser A., Kinsella C.M., Rueda P. (2020). Seasonal coronavirus protective immunity is short-lasting. Nat. Med..

[bib13] Galipeau Y., Siragam V., Laroche G., Marion E., Greig M., McGuinty M., Booth R.A., Durocher Y., Cuperlovic-Culf M., Bennett S.A.L. (2021). Relative ratios of human seasonal coronavirus antibodies predict the efficiency of cross-neutralization of SARS-CoV-2 spike binding to ACE2. EBioMedicine.

[bib14] Grifoni A., Weiskopf D., Ramirez S.I., Mateus J., Dan J.M., Moderbacher C.R., Rawlings S.A., Sutherland A., Premkumar L., Jadi R.S. (2020). Targets of T Cell responses to SARS-CoV-2 coronavirus in humans with COVID-19 disease and unexposed individuals. Cell.

[bib15] Grobben M., van der Straten K., Brouwer P.J., Brinkkemper M., Maisonnasse P., Dereuddre-Bosquet N., Appelman B., Lavell A.A., van Vught L.A., Burger J.A., Poniman M., Oomen M., Eggink D., Bijl T.P., van Willigen H.D., Wynberg E., Verkaik B.J., Figaroa O.J., de Vries P.J., Boertien T.M., Sikkens J.J., Le Grand R., de Jong M.D., Prins M., Chung A.W., de Bree G.J., Sanders R.W., van Gils M.J., Amsterdam UMC COVID-19 S3/HCW study group (2021). Cross-reactive antibodies after SARS-CoV-2 infection and vaccination. Elife.

[bib16] Hoffmann M., Krüger N., Schulz S., Cossmann A., Rocha C., Kempf A., Nehlmeier I., Graichen L., Moldenhauer A.S., Winkler M.S. (2022). The Omicron variant is highly resistant against antibody-mediated neutralization: implications for control of the COVID-19 pandemic. Cell.

[bib17] Hu B., Guo H., Zhou P., Shi Z.-L. (2021). Characteristics of SARS-CoV-2 and COVID-19. Nat. Rev. Microbiol..

[bib18] Huang A.T., Garcia-Carreras B., Hitchings M.D.T., Yang B., Katzelnick L.C., Rattigan S.M., Borgert B.A., Moreno C.A., Solomon B.D., Trimmer-Smith L. (2020). A systematic review of antibody mediated immunity to coronaviruses: kinetics, correlates of protection, and association with severity. Nat. Commun..

[bib19] Ieven M., Coenen S., Loens K., Lammens C., Coenjaerts F., Vanderstraeten A., Henriques-Normark B., Crook D., Huygen K., Butler C.C., GRACE consortium (2018). Aetiology of lower respiratory tract infection in adults in primary care: a prospective study in 11 European countries. Clin. Microbiol. Infect..

[bib20] Kang S., Yang M., He S., Wang Y., Chen X., Chen Y.Q., Hong Z., Liu J., Jiang G., Chen Q. (2021). A SARS-CoV-2 antibody curbs viral nucleocapsid protein-induced complement hyperactivation. Nat. Commun..

[bib21] Kundu R., Narean J.S., Wang L., Fenn J., Pillay T., Fernandez N.D., Conibear E., Koycheva A., Davies M., Tolosa-Wright M. (2022). Cross-reactive memory T cells associate with protection against SARS-CoV-2 infection in COVID-19 contacts. Nat. Commun..

[bib22] Ladner J.T., Henson S.N., Boyle A.S., Engelbrektson A.L., Fink Z.W., Rahee F., D’ambrozio J., Schaecher K.E., Stone M., Dong W. (2021). Epitope-resolved profiling of the SARS-CoV-2 antibody response identifies cross-reactivity with endemic human coronaviruses. Cell Rep. Med..

[bib23] Le Bert N., Tan A.T., Kunasegaran K., Tham C.Y.L., Hafezi M., Chia A., Chng M.H.Y., Lin M., Tan N., Linster M. (2020). SARS-CoV-2-specific T cell immunity in cases of COVID-19 and SARS, and uninfected controls. Nature.

[bib24] Leach S., Harandi A.M., Bergström T., Andersson L.M., Nilsson S., van der Hoek L., Gisslén M. (2021). Comparable endemic coronavirus nucleoprotein-specific antibodies in mild and severe Covid-19 patients. J. Med. Virol..

[bib25] Lin C.-Y., Wolf J., Brice D.C., Sun Y., Locke M., Cherry S., Castellaw A.H., Wehenkel M., Crawford J.C., Zarnitsyna V.I. (2022). Pre-existing humoral immunity to human common cold coronaviruses negatively impacts the protective SARS-CoV-2 antibody response. Cell Host Microbe.

[bib26] Lin D.-Y., Gu Y., Wheeler B., Young H., Holloway S., Sunny S.-K., Moore Z., Zeng D. (2022). Effectiveness of covid-19 vaccines over a 9-month period in North Carolina. N. Engl. J. Med..

[bib27] Lineburg K.E., Grant E.J., Swaminathan S., Chatzileontiadou D.S.M., Szeto C., Sloane H., Panikkar A., Raju J., Crooks P., Rehan S. (2021). CD8+ T cells specific for an immunodominant SARS-CoV-2 nucleocapsid epitope cross-react with selective seasonal coronaviruses. Immunity.

[bib28] Liu D.X. (2021). Annual review of microbiology similarities and dissimilarities of COVID-19 and other coronavirus diseases. Annu. Rev. Microbiol..

[bib29] Mateus J., Grifoni A., Tarke A., Sidney J., Ramirez S.I., Dan J.M., Burger Z.C., Rawlings S.A., Smith D.M., Phillips E. (2020). Selective and cross-reactive SARS-CoV-2 T cell epitopes in unexposed humans. Science.

[bib30] McLellan J.S., Chen M., Leung S., Graepel K.W., Du X., Yang Y., Zhou T., Baxa U., Yasuda E., Beaumont T. (2013). Structure of RSV fusion glycoprotein trimer bound to a prefusion-specific neutralizing antibody. Science.

[bib31] Ng K.W., Faulkner N., Cornish G.H., Rosa A., Harvey R., Hussain S., Ulferts R., Earl C., Wrobel A.G., Benton D.J. (2020). Preexisting and de novo humoral immunity to SARS-CoV-2 in humans. Science.

[bib32] Ortega N., Ribes M., Vidal M., Rubio R., Aguilar R., Williams S., Barrios D., Alonso S., Hernández-Luis P., Mitchell R.A. (2021). Seven-month kinetics of SARS-CoV-2 antibodies and role of pre-existing antibodies to human coronaviruses. Nat. Commun..

[bib33] Poston D., Weisblum Y., Wise H., Templeton K., Jenks S., Hatziioannou T., Bieniasz P. (2021). Absence of severe acute respiratory syndrome coronavirus 2 neutralizing activity in prepandemic sera from individuals with recent seasonal coronavirus infection. Clin. Infect. Dis..

[bib34] Russell M.W., Moldoveanu Z., Ogra P.L., Mestecky J. (2020). Mucosal immunity in COVID-19: a neglected but critical aspect of SARS-CoV-2 infection. Front. Immunol..

[bib35] Sagar M., Reifler K., Rossi M., Miller N.S., Sinha P., White L.F., Mizgerd J.P. (2021). Recent endemic coronavirus infection is associated with less-severe COVID-19. J. Clin. Invest..

[bib36] Schmidt F., Weisblum Y., Muecksch F., Hoffmann H.H., Michailidis E., Lorenzi J.C.C., Mendoza P., Rutkowska M., Bednarski E., Gaebler C. (2020). Measuring SARS-CoV-2 neutralizing antibody activity using pseudotyped and chimeric viruses. J. Exp. Med..

[bib37] Schulien I., Kemming J., Oberhardt V., Wild K., Seidel L.M., Killmer S., Sagar ., Daul F., Salvat Lago M., Decker A. (2021). Characterization of pre-existing and induced SARS-CoV-2-specific CD8+ T cells. Nat. Med..

[bib38] Sechan F., Grobben M., Edridge A.W.D., Jebbink M.F., Loens K., Ieven M., Goossens H., van Hemert-Glaubitz S., van Gils M.J., van der Hoek L. (2022). Atypical antibody dynamics during human coronavirus HKU1 infections. Front. Microbiol..

[bib39] Selva K.J., van de Sandt C.E., Lemke M.M., Lee C.Y., Shoffner S.K., Chua B.Y., Davis S.K., Nguyen T.H.O., Rowntree L.C., Hensen L. (2021). Systems serology detects functionally distinct coronavirus antibody features in children and elderly. Nat. Commun..

[bib40] Sikkens J.J., Buis D.T.P., Peters E.J.G., Dekker M., Schinkel M., Reijnders T.D.Y., Schuurman A.R., de Brabander J., Lavell A.H.A., Maas J.J. (2021). Serologic surveillance and phylogenetic analysis of SARS-CoV-2 infection among hospital health care workers. JAMA Netw. Open.

[bib41] Song G., He W.-T., Callaghan S., Anzanello F., Huang D., Ricketts J., Torres J.L., Beutler N., Peng L., Vargas S. (2021). Cross-reactive serum and memory B-cell responses to spike protein in SARS-CoV-2 and endemic coronavirus infection. Nat. Commun..

[bib42] Swadling L., Diniz M.O., Schmidt N.M., Amin O.E., Chandran A., Shaw E., Pade C., Gibbons J.M., Le Bert N., Tan A.T. (2022). Pre-existing polymerase-specific T cells expand in abortive seronegative SARS-CoV-2. Nature.

[bib43] Zhao J., Yuan Q., Wang H., Liu W., Liao X., Su Y., Wang X., Yuan J., Li T., Li J. (2020). Antibody responses to SARS-CoV-2 in patients with novel coronavirus disease 2019. Clin. Infect. Dis..

