# Supplementary material for: Recent infection with HCoV-OC43 may be associated with protection against SARS-CoV-2 infection
Source: iScience. 2022 Sep 9;25(10):105105. doi: 10.1016/j.isci.2022.105105 (PMC9458542; doi:10.1016/j.isci.2022.105105)
Supplement: Document S1. Figures S1–S3 and Tables S1–S4 [file mmc1.pdf]

iScience, Volume *25*

Supplemental information

Recent infection with HCoV-OC43 may be associated with protection against SARS-CoV-2 infection

A.H. Ayesha Lavell, Jonne J. Sikkens, Arthur W.D. Edridge, Karlijn van der Straten, Ferdyansyah Sechan, Melissa Oomen, David T.P. Buis, Michiel Schinkel, Judith A. Burger, Meliawati Poniman, Jacqueline van Rijswijk, Menno D. de Jong, Godelieve J. de Bree, Edgar J.G. Peters, Yvo M. Smulders, Rogier W. Sanders, Marit J. van Gils, Lia van der Hoek, and Marije K. Bomers

Supplemental Material

Table S1. Baseline characteristics by HCoV anti-NCt IgG lower versus highest levels; related to Table 1
Table S2. Availability of samples according to attendance to follow-up visit; related to Table 1
Table S3. Association between baseline Influenza- and RSV-antibodies and incidence of SARS-CoV-2; related to Table 2
Table S4. Baseline HCoV anti-S IgG and incidence of SARS-CoV-2; related to Table 2

Figure S1. HCoV-OC43 anti-NCt IgA; related to Table 2 (Figure S1A-B), Figure 1 (Figure S1C) and Figure 2 (Figure S1D)
Figure S2. Neutralizing antibodies in SARS-CoV-2 infected participants; related to Table 4
Figure S3. HCoV anti-S IgG over time in seronegative and seropositive participants; related to Figure 1

Table S1. Baseline characteristics by HCoV anti-NCt IgG lower versus highest levels, related to Table 1

Baseline characteristics	OC43 anti-NCt IgG		HKU1 anti-NCt IgG		229E anti-NCt IgG		NL63 anti-NCt IgG	
	Low (n=97)	High (n=32)	Low (n=98)	High (n=33)	Low (n=98)	High (n=33)	Low (n=95)	High (n=32)
Sex (%)								
Woman	75 (77.3%)	26 (81.3%)	75 (76.5%)	28 (84.8%)	78 (79.6%)	25 (75.8%)	74 (77.9%)	25 (78.1%)
Man	20 (20.6%)	6 (18.8%)	22 (22.4%)	4 (12.1%)	18 (18.4%)	8 (24.2%)	19 (20.0%)	7 (21.9%)
Unknown	2 (2.1%)	-	1 (1.0%)	1 (3%)	2 (2.0%)	-	2 (2.1%)	-
Age in years, median(IQR)	36.0 (27.0-47.8)	34.0 (26.5-46.5)	33.0 (26.0-46.5)	37.0 (29.3-49.3)	36.0 (26.3-48.8)	31.0 (27.0-45.0)	34.0 (26.0-47.0)	37.5 (30.0-49.3)
Living with children <12 years of age (%)	14 (14.4%)	3 (9.4%)	14 (14.3%)	3 (9.1%)	15 (15.3%)	2 (2.0%)	14 (14.7%)	3 (9.4%)
Unknown	30 (30.1%)	10 (31.2%)	30 (30.6%)	11 (33.3%)	30 (30.6%)	11 (33.3%)	30 (31.6%)	10 (31.2%)
SARS-CoV-2 serostatus (%)								
Positive	42 (43.3%)	6 (18.8%)	39 (39.8%)	9 (27.3%)	39 (39.8%)	9 (27.3%)	34 (35.8%)	13 (40.6%)
Negative	55 (56.7%)	26 (81.2%)	59 (60.2%)	24 (72.7%)	59 (60.2%)	24 (72.7%)	61 (64.2%)	19 (59.4%)
Disease severity (% of seropositive)								
No symptoms	15 (35.7%)	3 (50%)	14 (35.9%)	4 (44.4%)	14 (35.9%)	4 (44.4%)	13 (38.2%)	5 (38.5%)
Any symptoms	26 (61.9%)	3 (50%)	24 (61.5%)	5 (55.6%)	24 (61.5%)	5 (55.6%)	20 (58.8%)	8 (61.5%)
Unknown	1 (2.4%)	-	1 (2.6%)	-	1 (2.6%)	-	1 (2.9%)	-
Work-related exposure								
Bedside COVID-19 patient care	67 (69.1%)	19 (59.4%)	67 (68.4%)	21 (63.6%)	66 (67.3%)	22 (66.7%)	62 (65.3%)	23 (71.9%)
No patient care	30 (30.9%)	13 (40.6%)	31 (31.6%)	12 (36.4%)	32 (32.7%)	11 (33.3%)	33 (34.7%)	9 (28.1%)

Table showing characteristics for participants with highest quartile (high) and lower quartiles (low) of antibody levels against HCoV C-terminal domain nucleocapsid protein (NCt) at baseline.

Table S2. Availability of samples according to attendance to follow-up visit, related to Table 1

Available results per measurement, number of participants	OC43 anti-NCt IgG	HKU1 anti-NCt IgG	229E anti-NCt IgG	NL63 anti-NCt IgG	SARS-CoV-2 serostatus
March 2020 (% of total at visit)	129 (86.0%)	131 (87.3%)	131 (87.3%)	127 (84.7%)	150 (100%)
April 2020 (% of total at visit)	134 (95.0%)	136 (96.5%)	136 (96.5%)	132 (93.6%)	141 (100%)
May 2020 (% of total at visit)	137 (97.2%)	139 (98.6%)	139 (98.6%)	135 (95.7%)	141 (100%)
June 2020 (% of total at visit)	137 (97.9%)	139 (99.3%)	139 (99.3%)	136 (97.1%)	140 (100%)

Baseline samples were available for 132 participants; 6 participants had one or more unreliable results of HCoV anti-NCt IgG concentrations and these were therefore not included.

Table S3. Association between baseline Influenza- and RSV-antibodies and incidence of SARS-CoV-2, related to Table 2

Concentration at baseline	Low	High	Log-rank p-value	Univariable HR (95% CI)
Influenza hemagglutinin (HA)	34/97 (35.1%)	14/33 (42.4%)	0.42	1.29 (0.69-2.40)
RSV fusion protein (RSV-F)	35/97 (36.1%)	13/33 (39.4%)	0.68	1.44 (0.61-2.16)

Table showing the results of survival and cox regression analysis showing the association between the highest quartile (high) of Influenza HA and RSV-F versus three lower quartiles (low) at baseline (as a dichotomous determinant) and incidence of SARS-CoV-2 infection, as negative control analysis for Table 2.

Table S4. Baseline HCoV anti-S IgG and incidence of SARS-CoV-2, related to Table 2

HCoV anti-S IgG	All participants				Excluding participants with seropositivity at first measurement (March 2020)			
	Low	High	Log-rank p-value	Univariable HR (95% CI)	Low	High	Log-rank p-value	Univariable HR (95% CI)
OC43	38/98 (38.8%)	10/33 (30.3%)	0.33	0.71 (0.35-1.42)	13/73 (17.8%)	4/27 (14.8%)	0.68	0.79 (0.26-2.41)

HKU1	35/97 (36.1%)	11/32 (34.4%)	0.92	0.96 (0.49-1.90)	13/75 (17.3%)	3/24 (12.5%)	0.58	0.70 (0.20-2.46)
229E	34/98 (34.7%)	14/33 (42.4%)	0.29	1.40 (0.75-2.61)	15/79 (19.0%)	2/21 (9.5%)	0.33	0.49 (0.11-2.14)
NL63	36/98 (36.7%)	12/33 (36.4%)	0.95	0.97 (0.51-1.87)	14/76 (18.4%)	3/24 (12.5%)	0.49	0.65 (0.19-2.25)

Univariable survival and cox regression analysis showing the association between the highest quartile (high) versus lower quartiles (low) of antibody levels against spike (S) of HCoVs at baseline (as a dichotomous determinant) and incidence of SARS-CoV-2 infection, as comparison with analysis shown in Table 2. Analysis was repeated by excluding participants who already tested seropositive at the first measurement to correct for possible influence of elevated HCoV anti-S due to boosting upon SARS-CoV-2 infection.

Figure S1. HCoV-OC43 anti-NCt IgA, related to Table 2 (Figure S1A-B), Figure 1 (Figure S1C) and Figure 2 (Figure S1D)

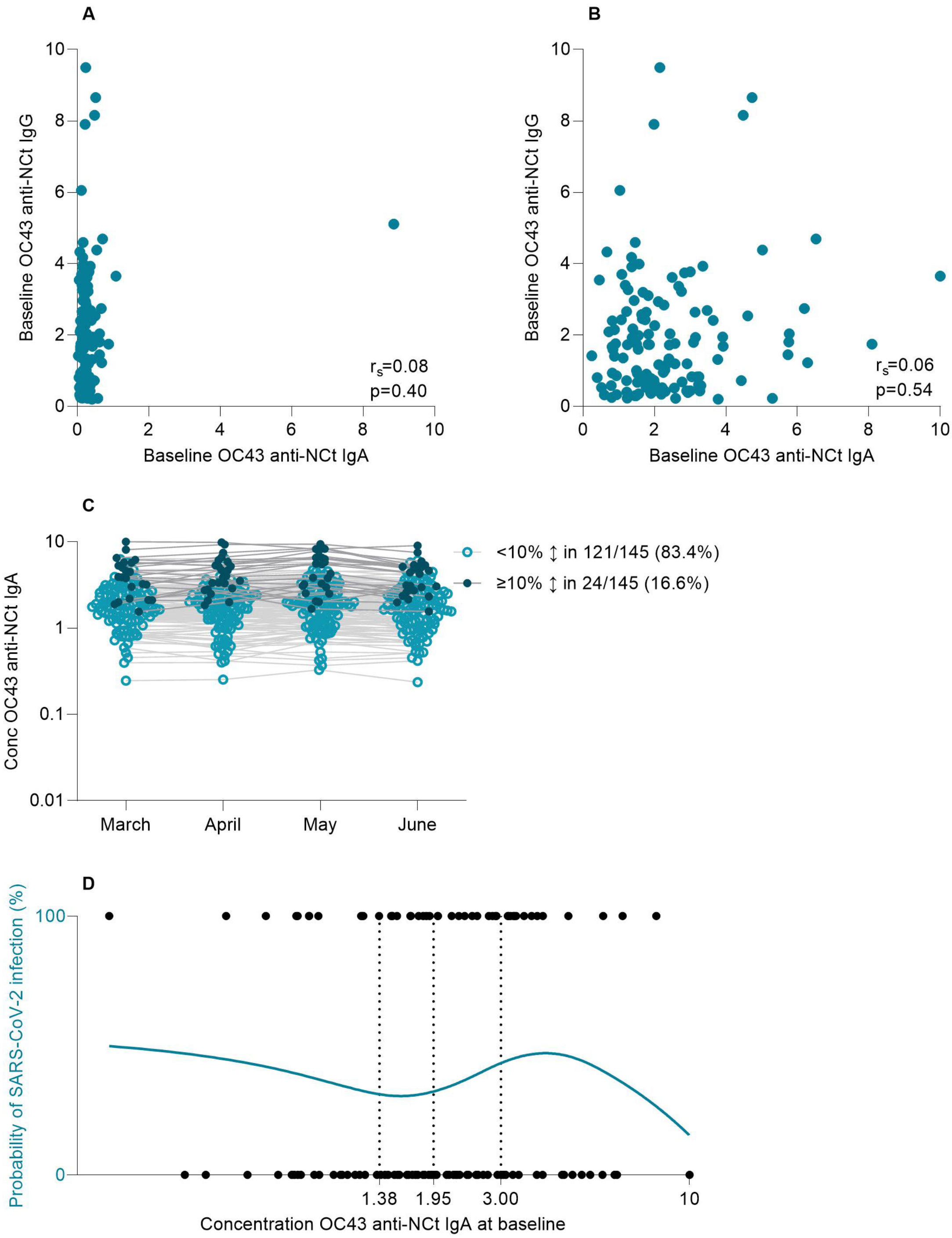

A: Scatter plot of correlation between baseline HCoV-OC43 anti-NCt IgG and IgA (n=128), assessed with Spearman's rank correlation coefficient. B: Scatter plot of correlation between baseline HCoV-OC43 anti-NCt IgG and IgA after exclusion of one outlier with very high levels of HCoV-OC43 anti-NCt IgA, assessed with Spearman's rank correlation coefficient. C: Scatter plot of HCoV-OC43 anti-NCt IgA over time (excluding one outlier). To determine fluctuation in antibody concentration, we calculated the difference between the highest and lowest concentration of each participant. A 10% difference in concentration equals 1 unit in standardized antibody concentration. D: Plot comparing HCoV-OC43 anti-NCt IgA (x-axis) excluding the aforementioned outlier, against SARS-CoV-2 status (right y-axis), with fitted binomial spline model with four knots represented by teal line; indicating the probability of seroconversion against SARS-CoV-2 (left y-axis).

Figure S2. Neutralizing antibodies in SARS-CoV-2 infected participants, related to Table 4

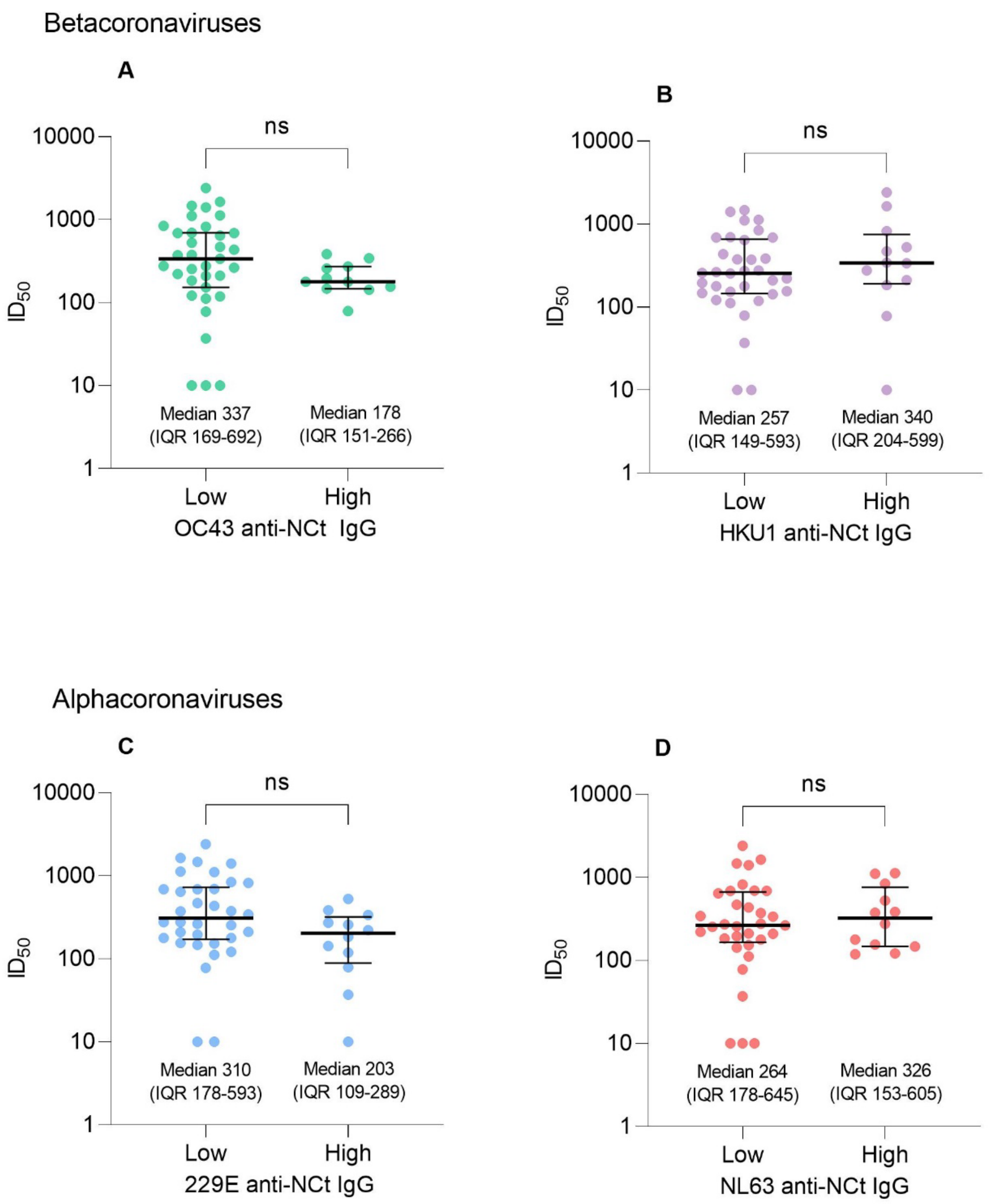

Plots individual values with median and interquartile range (IQR) of lower quartiles (low) versus highest quartile (high) of baseline HCoV anti-C-terminal nucleocapsid protein (NCt) IgG levels and neutralizing capacity (measured in June 2020) in log ID_{50} in SARS-CoV-2 seropositive participants. ns: not significant, assessed by Mann-Whitney U test.

Figure S3. HCoV anti-S IgG over time in seronegative and seropositive participants, related to Figure 1

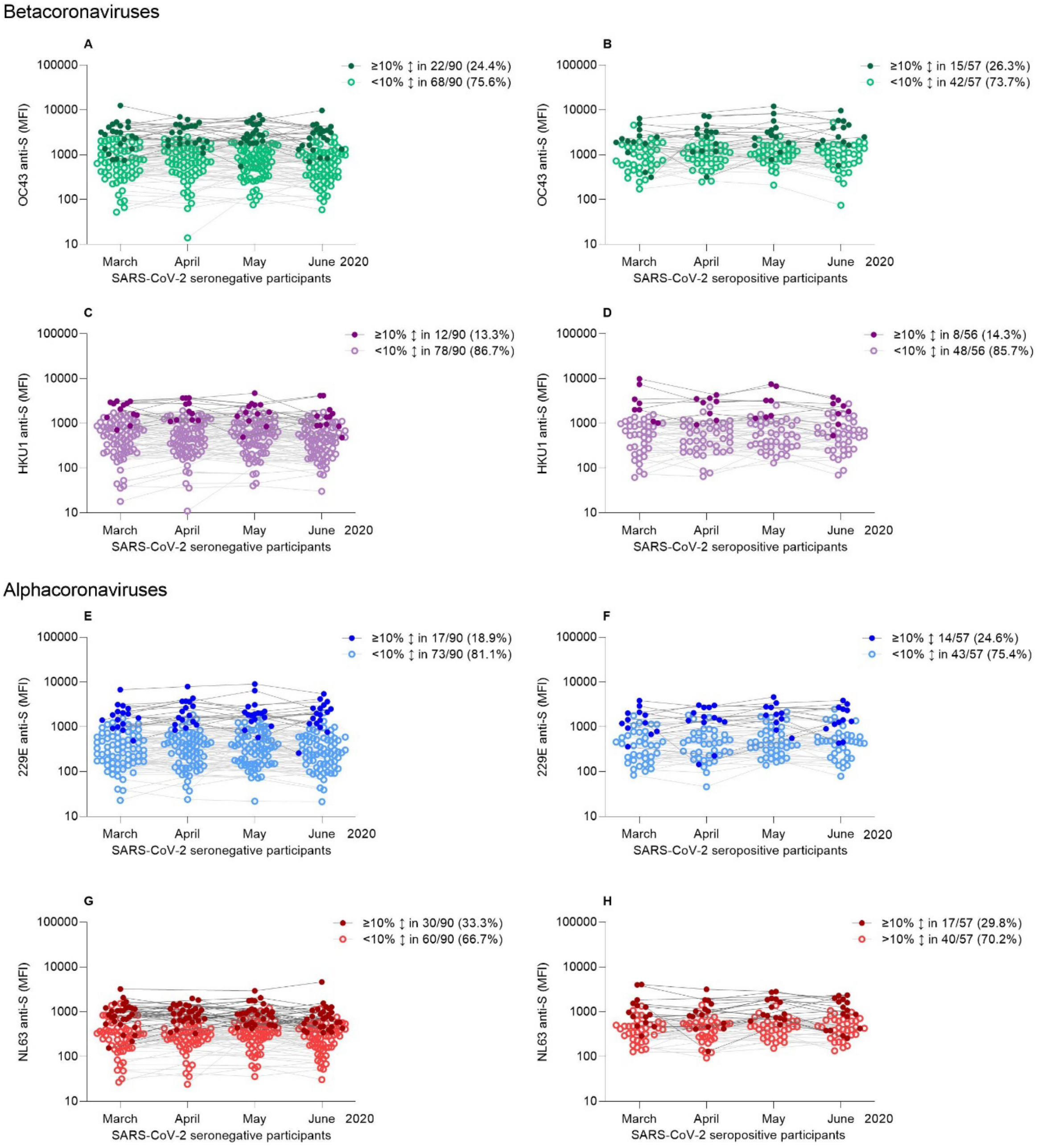

Scatter plots of HCoV spike (S) IgG levels over time, as a comparison with HCoV anti-C-terminal domain of nucleocapsid protein (NCt) IgG levels over time (depicted in Figure 1). To determine fluctuation in antibody concentration, we calculated the difference between the highest and lowest concentration of each participant and determined a 10% difference from the highest levels measured per HCoV.
